# Multiple-length-scale deformation analysis in a thermoplastic polyurethane

**DOI:** 10.1038/ncomms7583

**Published:** 2015-03-11

**Authors:** Tan Sui, Nikolaos Baimpas, Igor P. Dolbnya, Cristina Prisacariu, Alexander M. Korsunsky

**Affiliations:** 1 Department of Engineering Science, University of Oxford, Parks Road, Oxford OX1 3PJ, UK; 2 Diamond Light Source, Harwell Campus, Didcot OX11 0DE, UK; 3 Institute of Macromolecular Chemistry ‘Petru Poni’, Aleea Grigore Ghica Voda, Number 41A, Iasi 700487, Romania

## Abstract

Thermoplastic polyurethane elastomers enjoy an exceptionally wide range of applications due to their remarkable versatility. These block co-polymers are used here as an example of a structurally inhomogeneous composite containing nano-scale gradients, whose internal strain differs depending on the length scale of consideration. Here we present a combined experimental and modelling approach to the hierarchical characterization of block co-polymer deformation. Synchrotron-based small- and wide-angle X-ray scattering and radiography are used for strain evaluation across the scales. Transmission electron microscopy image-based finite element modelling and fast Fourier transform analysis are used to develop a multi-phase numerical model that achieves agreement with the combined experimental data using a minimal number of adjustable structural parameters. The results highlight the importance of fuzzy interfaces, that is, regions of nanometre-scale structure and property gradients, in determining the mechanical properties of hierarchical composites across the scales.

## Abstract (web summary)

Thermoplastic polyurethane elastomers are of interest in many applications, but their structure–property relations are not well understood. Here, the authors perform a multiple-length-scale deformation analysis on a thermoplastic polyurethane and shed light on morphological changes during the deformation.

## Graphical abstract

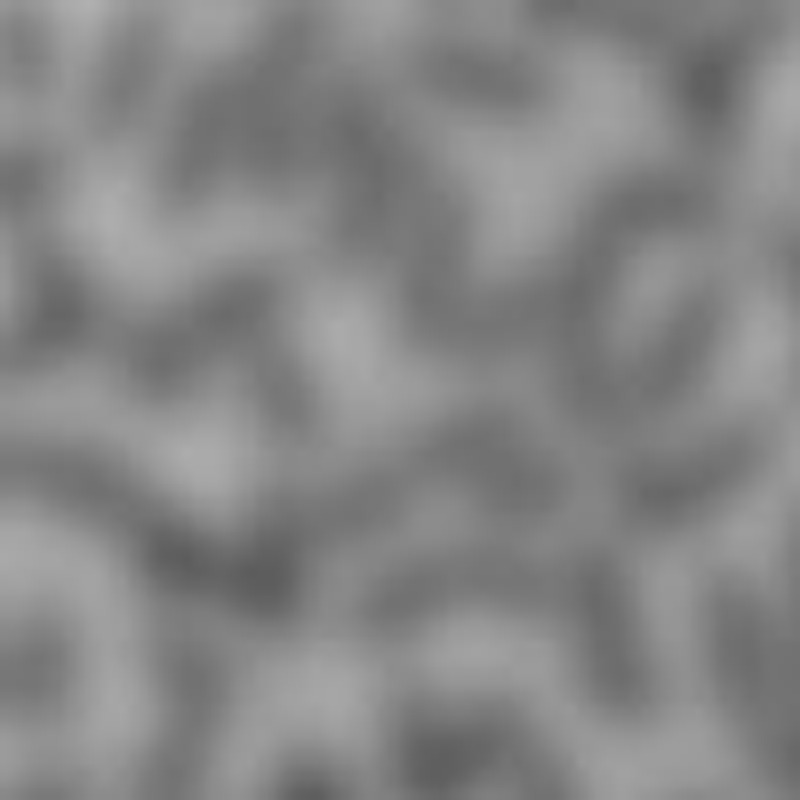

Thermoplastic polyurethane elastomers (TPUs) cover an extremely wide range of applications today due to their remarkable properties and versatility. These block co-polymers are composed of rubbery soft segments (SSs) and rigid hard segments (HSs)^1^,^2^. TPUs are an important class of two-phase elastomers in their own right, and a better understanding of their behaviour is still badly needed in the context of generic interest in the constitutive response of nano-structured composites.

The high sensitivity of the structure of this kind of material to the chemical composition, and synthesis and processing routes highlight the importance of understanding the way in which the structure determines the performance of TPUs. It is widely accepted that the superior properties of the TPUs are directly related to their multi-phase microstructure, in which the hard domains act as reinforcing filler connected by thermally reversible crosslinks and embedded in the soft matrix^1^,^2^. However, there is considerably less clarity and consensus regarding the existence and role of regions intermediate between the SSs and HSs, both in terms of the location and their mechanical properties, such as stiffness and hardness; and also their significance for the mechanical response of TPUs under monotonic and repeated loading.

There have been many attempts to explain the TPUs inelastic features, and to capture them in constitutive models. In the present work, this problem was addressed by studying a particular model TPU with HSs that were generated from the model 4,4′-methylene bis(phenyl isocyanate) (MDI).

The purpose of the present study is to review critically the prevailing binary hard–soft view of TPU structure, and to propose a model that takes into account the continuous variation of density and stiffness between the hard and soft phases. We also show that only such modified view of the TPU structure is consistent with the experimental observations using wide- and small-angle X-ray scattering (WAXS/SAXS).

The deformation behaviour of TPUs has been investigated extensively using a variety of experimental techniques^3^,^4^,^5^. However, despite the appreciation that the macroscopic properties are determined by the micro- and nano-scale structure, the relationship between the multi-phase nature of these materials and deformation inhomogeneity across the scales has not been fully explored and understood. The present investigation was conducted to address the clear need for a combined systematic experimental and modelling study of the structure-mechanical relationship that can provide a rational approach to the elucidation of the physical deformation mechanisms of TPUs.

Synchrotron-based X-ray imaging and X-ray scattering analysis are a family of powerful techniques for the study of material structure and deformation. Such is the versatility of these methods that literally all length scales of interest can be interrogated, often using the same X-ray beam settings. Full-field imaging resolution with a pixel size of ~1 μm can be achieved routinely using scintillator-coupled imaging detectors. Post-processing using Digital Image Correlation allows deformation analysis at the micron to millimetre scales. SAXS is an advanced non-destructive diffraction technique widely used to obtain quantitative structural information in crystalline and amorphous materials at the nano-scale. Of particular interest for the present study is the implementation of this technique for the observation of *in situ* deformation of TPUs^6^,^7^. One aspect of SAXS analysis that appears to be lacking is the ability to model diffuse interfaces and their effects on the direct and inverse analysis of diffraction data. Finally, synchrotron-based WAXS is widely used to quantify the crystallographic properties and crystal lattice internal strain in response to the external load. The simultaneous use of SAXS/WAXS has recently been applied to evaluate the structural evolution of polyurethane and other elastomers^8^,^9^,^10^.

The thorough interpretation and understanding of the SAXS/WAXS data collected in the reciprocal space allows direct comparison and validation against high-resolution transmission electron microscopy (TEM) or atomic force microscopy imaging in real space. Ultimately, the insights obtained on the basis of real and reciprocal space analytical methods must be expressed in the form of morphological deformation models that match the combined experimental observations^6^,^9^.

In the present study, an *in situ* synchrotron-based approach combining WAXS, SAXS and radiographic imaging is used to provide new insights into the morphological changes and deformation behaviour of TPUs during deformation. The strain factors for multiple-length-scale deformation analysis are determined. The measured multiple-length-scale strains of the TPU sample subjected to external tensile loading are validated by comparison with image-based finite element modelling (FEM) coupled with fast Fourier transform (FFT) analysis. Only three adjustable structure-related parameters are introduced in the simulations to describe the stiffness ratio of the HSs to SSs, the volume fraction of the soft phase, and the sharpness of the transition between the two phases. The multi-phase nature and the continuous property variation of polyurethane are incorporated in the model, providing a means of explaining the observed relationships between the strains measured at different scales.

## Results

### Multiple-length-scale strain analysis

**Figure 1:**
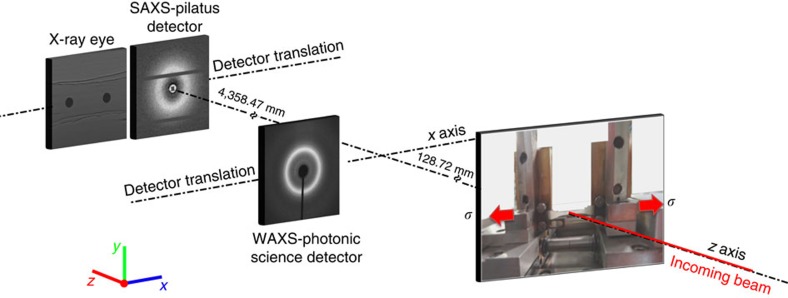
Schematic diagram of the experimental set-up. The sample was subjected to repeated uniaxial loading within the tensile stage. Radiographic images and SAXS and WAXS patterns were recorded at each loading step. After collecting each radiographic image, the SAXS detector was placed in the beam to collect SAXS patterns, and then WAXS detector was translated into the beam to collect XRD data.

Schematic diagram of the experimental set-up shown in Fig. 1 is described in detail in the Methods section.

**Figure 2:**
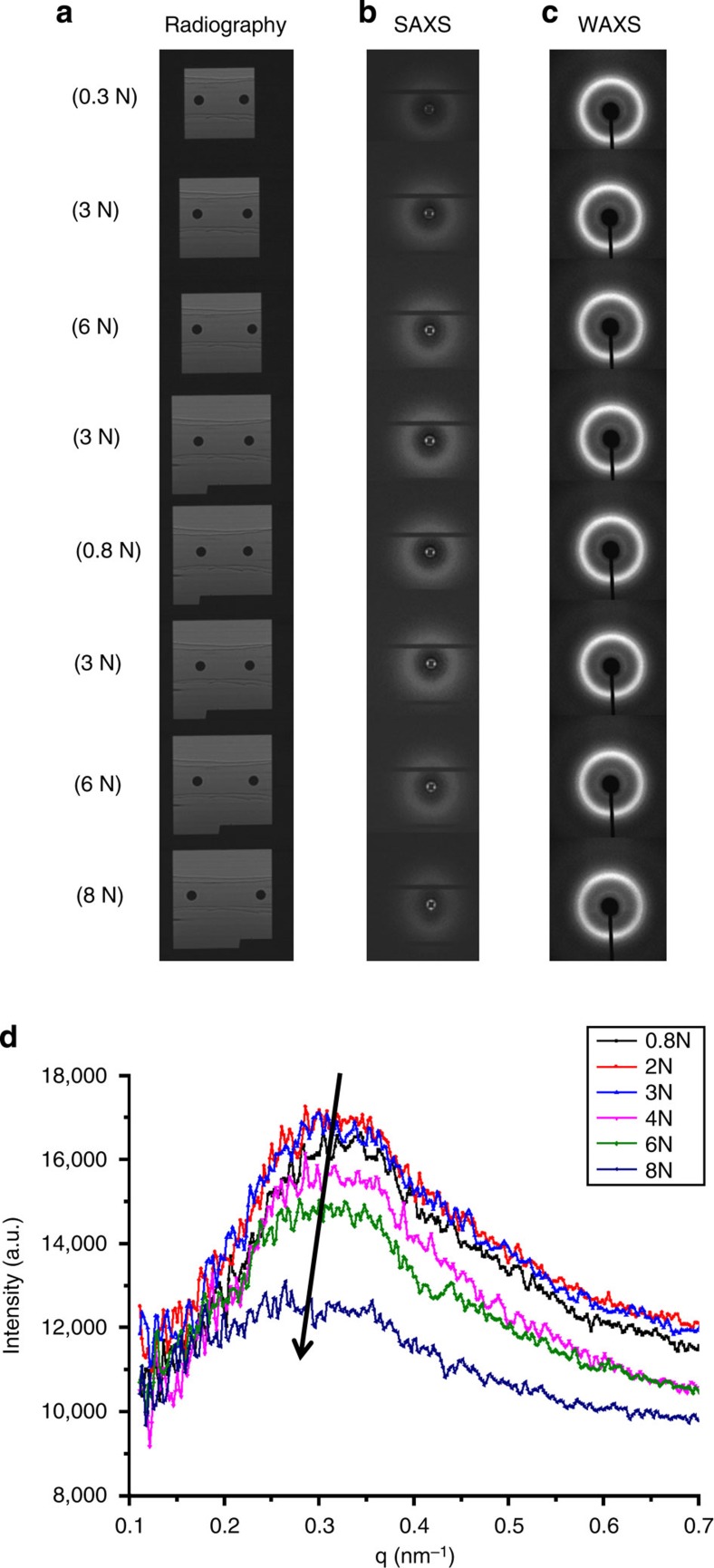
Hierarchical structural evolution of 2D X-ray images and diffraction patterns. (**a**) Selected radiographic images acquired by the ‘X-ray Eye’ detector, and (**b**) SAXS and (**c**) WAXS patterns of TPUs during repeated loading, captured during the first loading stage from 0 to 6 N, then unloading stage from 6 to 0.8 N, and then the re-loading stage from 0.8 to 8 N. Each loading value is indicated in the label. The black line observed in **c** for each WAXS pattern is the beam stop. (**d**) The family of equivalent 1D profiles of scattered SAXS intensity (arbitrary units) versus *q* during monotonic loading between 0.8 and 8 N shows an appreciable peak shift towards lower *q* values, corresponding to the increase of linear dimension due to sample extension.

A dog-bone TPU sample was subjected to an *in situ* loading sequence (0 N, 6 N, 0.8 N, 8 N), and its structural evolution was analysed using radiographic imaging, and SAXS and WAXS pattern interpretation, as illustrated in Fig. 2. The sample was slowly deformed along the *x* axis in the laboratory coordinate system at a displacement rate of 0.5 mm min^−1^, using a remotely operated and monitored rig (Deben, Suffolk, UK) equipped with a 150 N calibrated load cell. At each consecutive loading increment, the sample was held under constant load, while radiographic images and SAXS/WAXS patterns were collected. The projection of two small solder balls (0.5 mm diameter) glued on the surface of TPUs was captured radiographically as shown in Fig. 2a. The loading values appear in the columns as labels next to the images, indicating that the images were first collected in a sequence during loading up to 6 N, then during unloading to 0.8 N, and finally during re-loading up to 8 N.

Figure 2b presents the evolution of two-dimensional (2D) SAXS patterns of the TPU sample. The patterns showed minimal change during the first loading cycle (0 N, 6 N, 0.8 N), and then were seen to evolve gradually from circular to elliptical during re-loading up to 8 N. The preferential orientation was seen to become aligned with the *x* axis, i.e., the short axis of the elliptical pattern in reciprocal space corresponding to the direction of extension in real space. Fig. 2c shows the corresponding WAXS patterns consisting of a system of diffraction rings (peaks). The evolution behaviour as a function of load that was similar to that of the SAXS patterns is also observed. It is also noted that the pattern segments close to the *y* axis gradually became brighter, indicating the development of orientation anisotropy associated with preferred alignment of crystallites. Fig. 2d shows the equivalent radial line profiles obtained from the SAXS patterns.

**Figure 3:**
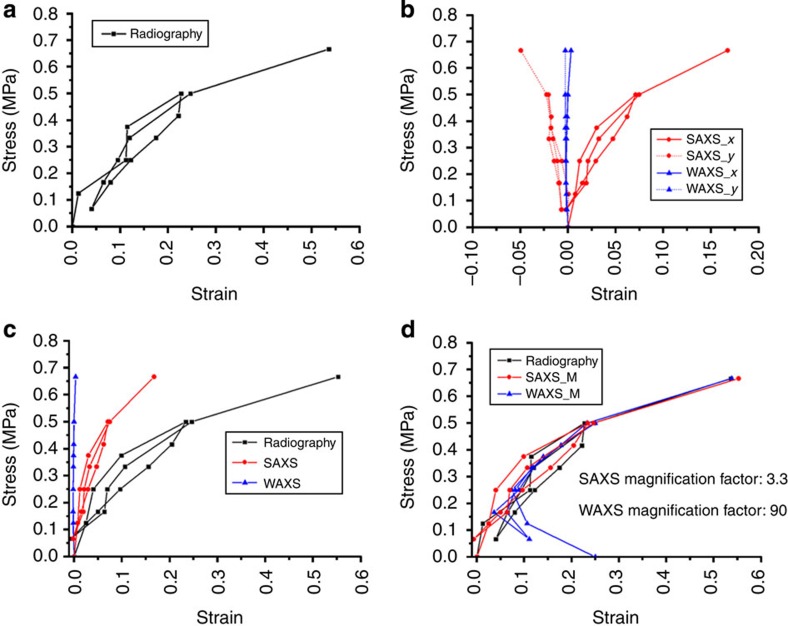
Multiple-length-scale strains visualization. (**a**) Evolution of the macroscopic longitudinal strain measured by radiography X-ray eye. (**b**) Evolution of the nano-scale and sub-nano-scale strains interpreted from SAXS/WAXS patterns along both longitudinal *x* (solid) and transverse *y* axes (dash). The negative transverse strains reveal the Poisson effect that was observed in the experiment. The comparison of the macroscopic strain, sub-nano-scale strain and nano-scale strains along the loading direction (*x* axis) was shown in **c** and **d**, where **c** shows the original results and **d** shows the modified results.

From the radiographic images (Fig. 2a) obtained using the Photonic Science sCMOS detector (sometimes referred to as the ‘X-ray Eye’), the macro-strain evolution was quantified by examining the relative elongation of the ligament between two solder balls as illustrated in Fig. 3a. In Fig. 3c, these strains were plotted together with the nano-scale strains calculated from the interpretation of the SAXS patterns (see Fig. 2d), and at the crystal lattice-scale strain from WAXS pattern analysis (Fig.2c). The strains along the loading direction (*x* axis) and transverse to it (*y* axis) were plotted as abscissa, with the applied load as ordinate in Fig. 3b. The presence of the Poisson effect is manifested in the relative magnitude and sign of the strains along the two axes.

Figure 3c highlights the significant difference in the strain magnitude depending on the length scale of consideration. The macroscopic strains along the loading direction (*x* axis) are significantly greater than the SAXS-derived nano-scale strains, which, in turn, are much greater than the elastic lattice strains calculated on the basis of WAXS data. The ratio of the macroscopic-, nano- and lattice-scale elastic strains along the loading direction was approximately given by


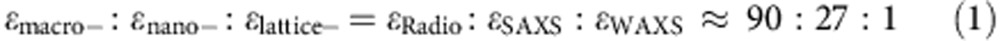


Of particular note is the fact that the above ratio remained substantively unchanged during loading, unloading and subsequent re-loading of the sample. To demonstrate this fact clearly, the stress–strain plots shown in Fig. 3d have been modified by magnifying the nano-scale and lattice-scale strains by the appropriate scaling factors to bring them ‘in line’ with the macroscopically observed strains. The nano-scale (SAXS) strain was multiplied by the factor of 90/27=3.3, while the lattice-scale (WAXS) strain was multiplied by the factor of 90/1=90. The observed good match after the modification confirms that the strain scaling factors given in equation (1) remain unchanged during deformation.

### Image-based FEM modelling and FFT analysis

To aid the understanding of the origins and the meaning of the strain factors between the multiple-length scale measurements (macroscopic, nano-scale and lattice-scale), the deformation of polyurethane elastomer was simulated numerically by the combination of FEM and FFT analysis.

**Figure 4:**
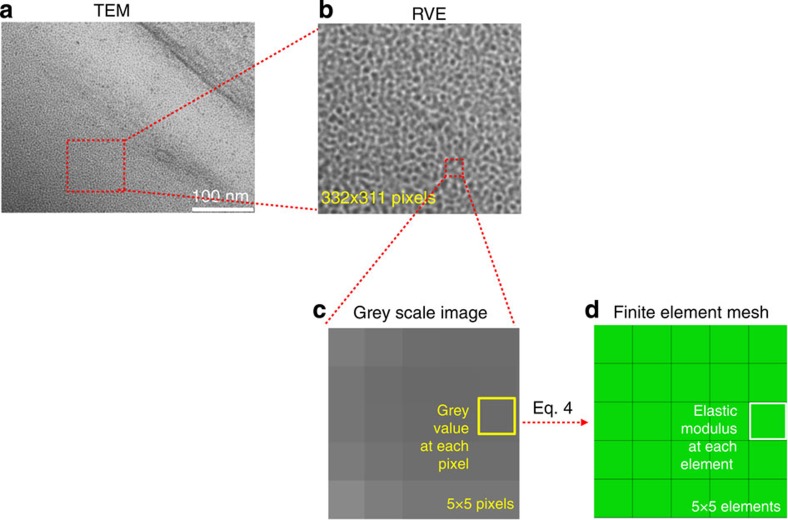
Image-based FE modelling. (**a**) The original TEM image. (**b**) A representative volume element (RVE) with 332 × 311 pixels was selected from **a.** (**c**) Representative 5 × 5 pixels (elements) were chosen to demonstrate the modelling procedure. Each pixel in **c** corresponds to an element in (**d**) the finite element model generated and meshed in ABAQUS.

An illustration of the internal nano-scale structure of a block co-polymer is provided by the TEM image of a similar TPU shown in Fig. 4a TPU (courtesy of Dr Kayleen Campbell, University of Queensland). Fig. 4b shows a magnified bright-field TEM image of a thin lamella (<100 nm) of this material. The perceived pixel brightness in transmission TEM is closely related to the electron density of the illuminated material volume. Therefore, the TEM image represents a density map that reveals the nano-scale gradients and inhomogeneities of material density and related stiffness. The darker areas seen in the TEM image are associated with dense, hard regions that act as the primary contributors to WAXS scattering. In contrast, the lighter areas in the TEM image correspond to less dense, more compliant regions that are likely to be associated with tie molecules that maintain connections between denser regions. It is important to note the presence of gradients of density between these regions that affect in equal measure the deformation and scattering behaviour of the material.

The development of the numerical models described below proceeds on the basis of the following assumptions. On the basis of the TEM image of a thin lamella, a 2D FE model is created that is suitable for considering the consequence of applying remote macroscopic stress, and computing the strain values at the macro-, nano- and angstrom-length scales. This allows the macro- and angstrom-length scale strains to be extracted directly from the model. However, the evaluation of the nano-scale strain requires the FEM results to be post-processed as follows. First, the SAXS pattern from the thin lamella must be obtained by performing 2D Fourier transform, using the square of the amplitude of the propagated wave to compute the intensity as a function of the scattering angle. Following azimuthal binning, the 2D pattern are converted into the equivalent 1D profile (intensity versus *q*). The *q* value that gives the highest intensity is then found by peak fitting, and from this value the nano-scale distance measure is extracted and converted to strain.

This SAXS post-processing analysis reflects the statistics of each thin lamella deformation. The ‘thick’ sample used in the *in situ* X-ray experiment can then be considered as a ‘stack’ of multiple thin lamellae. Since the overall SAXS pattern arises from intensity superposition of multiple thin lamellae, and as these are considered to be statistically identical, the model is fully representative of the experimental X-ray scattering conditions.

Image-based modelling was used to generate structure-specific multi-phase models of TPU samples as follows. A representative volume element (RVE) with 332 × 311 pixels taken from the original greyscale TEM image (Fig. 4a) was selected (Fig. 4b). Each pixel corresponded to a square element centred at the corresponding coordinate, giving rise to a model composed of 332 × 311 elements, each having identical geometry, but varying properties. The model was implemented in the form of a finite element mesh in ABAQUS. An example of 5 × 5 pixels (elements) is shown in Fig. 4c,d to demonstrate the modelling procedure. The continuous property variation was captured by means of introducing multiple ‘pseudo-phases’. The grey value of the particular pixel was used to compute the corresponding modulus for each element.

A modulus-density function was proposed to describe the relationship between the grey value of a pixel in Fig. 4c and the corresponding elastic stiffness assigned to the element in Fig. 4d. The nature of the function and the reason for choosing this relationship are described in the Methods section. The initial values of material parameters were taken from the literature^11^: the elastic modulus of the HS of amorphous polymers was taken to be equal to ~1–3 GPa, and the effective modulus of the rubbery polymer (SS) was taken to be ~1–50 MPa, respectively. The volume fraction of the HS was taken to be ~35–40%.

SAXS pattern simulation was sequentially coupled to FE deformation modelling. The scattering process described by the Fourier transform from the ‘real’ (laboratory) space to the ‘reciprocal’ space of scattering vectors. Diffuse elastic scattering of the primary beam is due to the coherent scattering from electrons, that is, mutual interferences among electromagnetic waves scattered from different positions within the gauge volume. The principle can be explained by considering the X-ray beam impinging on the sample containing a particular distribution of electron density. The measured scattering intensity *I*(*q*) is related to the squared modulus of the Fourier transform of the electron density distribution *ρ*(*r*) as follows:


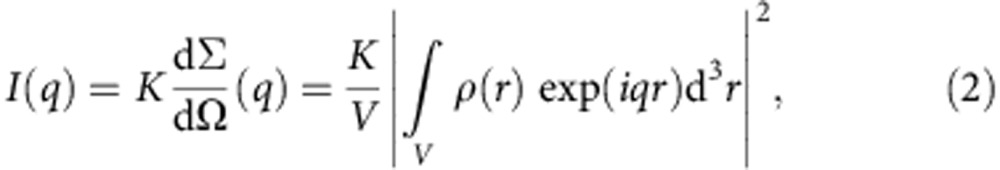


where *V* is the sample volume, *K* is an instrumental constant and 
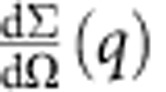
 corresponds to the macroscopic differential scattering cross-section of the sample^12^.

**Figure 5:**
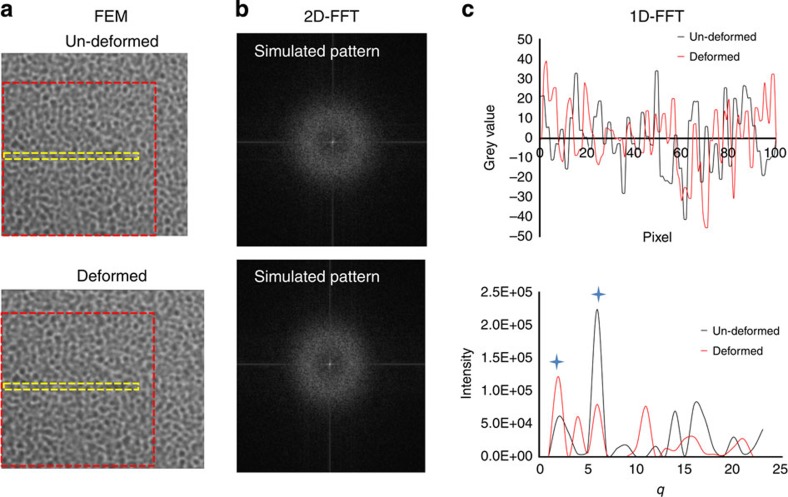
Results of image-based finite element modelling and FFT analysis of TPUs. (**a**) The same greyed values from the RVE image were assigned on the finite element model. Both the un-deformed and deformed shapes were shown. (**b**) Simulated 2D SAXS patterns of the selected region marked by a red square in **a** using FFT. (**c**) The 1D FFT analysis of electron density evolution along *x* axis in the selected region marked by a yellow rectangular in **a**.

The grey-scale model of the material before and after deformation simulation by ABAQUS is illustrated in Fig. 5a. To perform the direct calculation of the SAXS pattern from TPU, a consistent fixed region of interest was selected from both the un-deformed and deformed images in Fig. 5a. The region is marked by a red square, and notionally corresponds to the beam spot on the sample. The corresponding SAXS patterns were computed by 2D FFT and are shown in Fig. 5b. In correspondence with our interpretation of experimental data, the 2D SAXS patterns were processed into 1D radial plots by integrating over the ring pattern within the azimuthal angle range of ±10°. Then the nano-scale strain along the loading direction was quantified by examining the displacement of the peak centre in the SAXS patterns. The detail is described in the Methods section.

A 1D electron density peak profile of the selected line region in Fig. 5a (marked in yellow rectangular) is shown in Fig 5c. A shift in the peak position is observed, with the most prominent peak indicated by the blue cross markers. Thus, the FE model post-processing by means of SAXS pattern simulation provides direct evidence of the way the electron density variation during deformation results in the peak shift in reciprocal space.

In setting up the simulation, we are seeking to resolve the following problem. The approach often taken in analysing SAXS data is limited to looking at the variation of the structural period in the range of length scales accessible for small-angle scattering analysis. Let us consider the deformation of a simple two-phase material consisting solely of HSs and SSs with sharp interfaces. For simplicity, we may picture the material as a 1D, periodic line of HSs and SSs. When this line is subjected to stretching, the HSs and SSs deform to different strains. However, due to the periodic nature of the model and the assumption that SAXS pattern is only sensitive to the distance between sharp interfaces (jumps of electron density), it is easy to see that the deformation ends up being affine, and the overall strain of a structural period must be equal to the macroscopic strain.

We surmise that (a) SAXS pattern formation depends on the gradual, smooth transitions in electron density and (b) inhomogeneous deformation results in changes in the electron density gradients, which in turn affects the apparent position of the peak observed in the SAXS pattern. To test this hypothesis, we undertake numerical simulations and seek to match the experimentally observed nano-scale strain ‘deficit’ in comparison with the macroscopic value.

The macroscopic strain (*ε*_external_) was simulated by applying a similar displacement boundary condition to the whole finite element model. The sub-nano-scale strain distribution was collected by averaging the strain of the HSs in the model, which had lower grey values, along the loading direction (*ε*_*xx*_). The nano-scale strain value (*ε*_FFT_) was determined by looking at the peak movement of the simulated pattern (Fig. 5c) by processing the FFT of the un-deformed and deformed patterns (Fig. 5a) exported from ABAQUS. By refining the values of *a* and *b* in Equation 4, the best fitting that could be achieved was


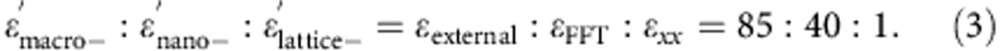


The final parameter values found by refinement were *a*=90 and *b*=0.1.

The ratios of characteristic length scales of analysis and of the strains measured at each of these length scales depend on the specific nature of the gradient nano-composite studied. However, the review of published literature [6,7,14] suggests that the ratio of SAXS-derived nano-scale strain to the macroscopic strain typically lies in the range 0.3–0.5. It is also clear that the ratio of the macroscopic strain to WAXS-derived strain at the lattice-length scale depends on the ratio between the stiffness of dense microstructural regions, on one hand, and the overall macroscopic stiffness of the composite.

## Discussion

In the course of the experiment, the data collection was performed as follows. The samples were loaded (or unloaded) to a particular level of applied stress, and held at constant stress value during data collection. This mode of measurement is consistent with maintaining the elastic part of internal strain as close to constant as possible during acquisition. The viscous effects during data collection were assessed by performing repeated measurements under constant applied stress. Since this revealed no appreciable changes in the scattering patterns, deformation simulation on the basis of elastic modelling was considered appropriate. It is worth re-iterating here that the primary aim was to resolve the fundamental relationship between multi-scale deformation and apparent X-ray scattering. This relationship is rooted in the solution of the steady-state continuum mechanics problem that must satisfy the stress equilibrium and strain compatibility at all scales. The model did not attempt to predict the entire time-dependent or cyclic visco-elastic response, but focused on capturing the multi-scale strain relationship. Repeated loading, unloading and re-loading was performed to exclude the possibility of the development of internal residual stresses that may affect the interpretation. It was observed that the second re-loading of material followed a stress–strain path closer to the previous unloading path than to the first loading path^11^. The effects of path variation during unloading and re-loading, however, were seen to be moderate: the re-loading curves for macrostress versus micro- or nano-strain appear equidistant from both the original loading curve and the unloading curve. Multiple loading cycles were essential for assessing the consistency of the multiple-length-scale strain ratios.

To carry out numerical simulation of the deformation behaviour of a nano-structured block co-polymer, we require a description of the spatial variation of material stiffness. Recapitulating the well-known fact that in bright-field TEM images, regions of high electron density appear dark, whereas regions of low electron density appear bright^13^, we adopt a simple assumption that, since stiffness is also related to the local (electron) density, a function can be formulated that provides an expression for the local stiffness in terms of the grey value of the TEM image. The quantitative relationship is described by equation (4) given in the Methods section. The function contains adjustable parameters that describe the ratio between the extreme values of stiffness (hard/soft), as well as the steepness of the transition between them, and the volume fraction of the hard phase (*a*, *c* and *b*). The ratio of modulus between the hard region and soft region, *a*, mostly affects the ratio 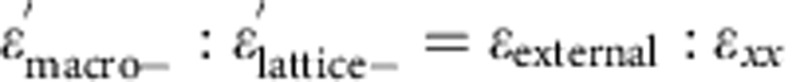. As in the classical composite theory for the hard inclusions embedded in the soft matrix (for example, the Eshelby inclusion model^14^), the relationship between the macro-strain and the inclusion strain is inversely correlated to the corresponding ratio of modulus between the two phases. The values of parameters *b* and *c* control the steepness of the local stiffness gradients in the transition regions between the hard and soft elements, and hence the resulting deformation distribution. Therefore, *b* and *c* control the ratio 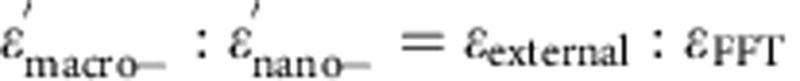. The selection of parameter values is explained in the Methods section. Good agreement achieved between the model and experiment indicates that simultaneous refinement of model parameters allows capturing correctly the physical mechanisms responsible for the observed strain ratios.

The ratio of the nano-scale strain (by SAXS) to macro-strain has been reported to lie in the region 0.3–0.5 for uniaxial loading^15^, which is roughly consistent with the present finding (27/90=0.3). Different values of this ratio may result from distinctly different internal nano-scale structures and continuous electron density distributions in different types of polyurethane.

**Figure 6:**
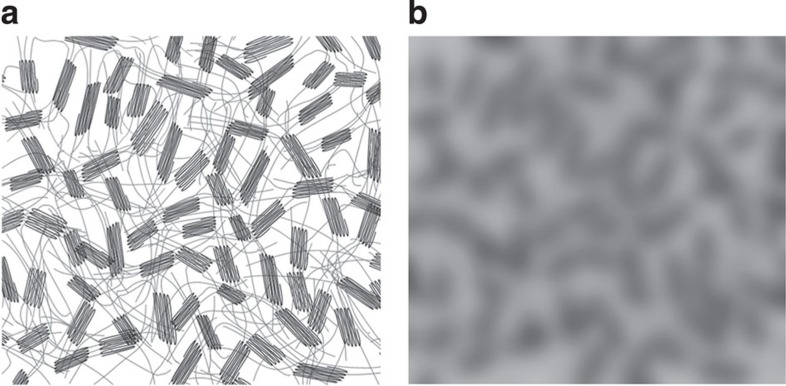
Fuzzy interfaces. (**a**) Schematic view of the TPU nanostructure and (**b**) the average electron density image obtained by ‘blurring’.

The observation that the strain ratios remain the same during repeated loading brings into question the rotation and fragmentation model, since fragmentation cannot be reversed during unloading^6^. Instead, we propose a multi-scale view of TPU deformation that is illustrated in Fig. 6. The HSs are characterized by the presence of polar side chains experiencing strong attraction that leads to so-called physical cross-linking, resulting in a high degree of aggregation and order, forming regions that can be crystalline or pseudo-crystalline, depending on specific chemistry. These strongly aggregated regions are embedded in a soft and compliant matrix formed by the more flexible, low polarity segments. In the vicinity of hard aggregated areas, the flexibility of soft chains is sterically and conformationally restricted, leading to a gradual increase in the local stiffness from the low value characteristic of the SSs towards that of the HSs, on the length scale of a few nanometres. In the course of extensional deformation nanometre-long lengths of polymer chain may become pulled out of the HSs, making an additional contribution to the gradual hard–soft transition. Interestingly, the above process may be completely or partially reversible upon removal of the tensile load.

In Fig. 6a, the HSs are schematically drawn as multiple-folded lines. Figure 6b is obtained by blurring Fig. 6a to obtain a simulated TEM image with the resolution of a few nanometres. Following this transformation, the HSs correspond to the darker regions associated with the high electron density in Fig. 6b. In contrast, the more flexible matrix around the crystalline hard regions that appears as random connecting lines between the HSs in Fig. 6a, corresponds to the regions of significantly lower electron density (lighter grey) in Fig. 6b. It is the transition between these regions that is of the greatest interest to the present study. The transition from the soft amorphous matrix to the hard region is gradual and corresponds to progressively improving density of physical cross-linking. During deformation some lengths of the chain at the edges of the highly cross-linked (pseudo)crystalline region undergo pulling out into the softer matrix, leading to the reduction in electron density. This process is partly reversible: as the load is reduced, the restoration of some of the original stiffness is observed. The fact that this reversibility is incomplete means that the tangent stiffness at a given macro-strain during re-loading is lower than the original loading, but greater than that at the unloading stage.

In this study, the mechanical responses of polyurethane elastomers subjected to repeated 1oading were studied during *in situ* strain measurement by the combination of synchrotron radiography, SAXS and WAXS techniques. The multiple-length-scale strains were analysed by radiographic images at the macro-scale, SAXS patterns at the nano-scale and WAXS patterns at the sub-nanometric (crystal lattice) scale. The strain ratios between the multiple-length scales were found to be roughly 90:27:1 (lattice: nano: macro). The structure evolution of TPUs at different scales probed by multiple synchrotron X-ray imaging and scattering techniques during deformation were simulated and validated by TEM image-based FEM coupled with 2D FFT analysis. Only three parameters were included in the TPU structural description function that postulated the relationship between the pixel grey values in the TEM images and the local elastic modulus used in the ABAQUS FE simulation. Good agreement between the measured and calculated multi-scale strain was achieved by adjusting the function parameters.

The systematic experimental and modelling approach presented in this paper represents the introduction of physically based modelling approach to the analysis of reciprocal space (SAXS/WAXS) data. The correlated multiple-length-scale strain evolution analysis opens up the route towards better insights into the structure–property relations for structural polymers such as TPUs. This knowledge is essential for the future development and molecular design of TPUs with high functionality and improved performance.

## Methods

### Sample preparation

The model TPU for this work was produced in the IMC laboratory (Iasi, Romania). It was synthesized as a three-component system combined in stoichiometric proportions, consisting of: (1) a di-isocyanate—MDI; (2) a MD (macrodiol) polytetrahydrofuran of molar mass Mw=2,000 g mol^−1^; and (3) a small molecule diol as CE (chain extender)—anhydrous diethylene glycol. The three components were mixed in the molar proportions HS:MD:CE=4:1:3, giving HS mass fraction of 40%, and isocyanic index *I*=100. Synthesis was carried out by the pre-polymer route described previously by Prisacariu *et al*.^16^,^17^,^18^ The DI and MD components were reacted together with vigorous mixing under vacuum at 100 °C, to give pre-polymer consisting of a macrodiol terminated at each end by di-isocyanate. This was then thoroughly mixed with the CE at 90 °C, and cast into a closed sheet mould for curing at 110 °C over 24 h. The final result was a polymer with Mw in the range 60–120 kg mol^−1^, in the form of a sheet with thickness 0.5–1.0 mm. The sheet was stored at room temperature for at least 1 month before testing. It should be noted that the stoichiometric proportions used in this polymer (*I*=100) means that this material is truly thermoplastic. It does not have the potential for further reaction with ambient humidity to produce chain lengthening and allophanate cross-linking, seen in similar polymers but with excess isocyanate groups (for example, *I*=110)^19^.

The polyurethane elastomer used for this study was designated ‘PU183’. All samples were cut into dog-bone shapes for the tension experiment, with a grip-to-grip length of 22 mm, a width of 3 mm at the centre of the gauge length and a thickness of 2 mm. Two solder balls were fixed symmetrically on the surface of each glued sample pair, whose movement can be tracked by X-ray Eye (Photonic Science X-ray MiniFDI) to capture the macroscopic deformation. In addition, the balls also served as the reference point for the location of X-ray beam on the centre of the otherwise transparent polymer sample.

### *In situ* X-ray scattering measurements

The experiment was performed on the B16 beamline at Diamond Light Source (DLS, Oxford, UK). The monochromatic X-ray beam at the photon energy of 17.99 keV was used and collimated to the spot size of 0.7 × 0.7 mm^2^. The beam was incident at the sample in the direction perpendicular to its plane and the loading direction. Two separate WAXS and SAXS detectors were alternately set up to collect the patterns at consecutive loading increments downstream of the beam. WAXS diffraction patterns were recorded using a Photonic Science Image Star 9000 detector (Photonic Science Ltd., UK) placed at a sample-to-camera distance of 128.72 mm. Further downstream, a Pilatus 300K detector (Dectris, Baden, Switzerland) was positioned at a distance of 4,358.47 mm to collect the SAXS patterns. Although only a uniaxial loading test was considered in this study, more complex loading conditions can also be addressed in a similar way. Note that the data contained in the WAXS and SAXS patterns allows the determination of the complete 2D strain state(s), including shear components.

In addition to diffraction detectors, an ‘X-ray Eye’ imaging detector (6.5 μm optical pixel size, Photonic Science Ltd. UK) was mounted besides the SAXS detector to capture the whole sample image during deformation. A lightly compacted disk of standard silicon powder and a dry chicken collagen sample inserted close to the sample position were used as calibration standards^20^,^21^, and to determine the sample-to-detector distance to good precision. The dog-bone-shaped sample was slowly deformed along the *x* axis in the laboratory coordinate under repeated uniaxial tensile loading (0 N, 6 N, 0.8 N, 8 N) at a displacement rate of 0.5 mm min^−1^, using a remotely operated and monitored compression rig (Deben, Suffolk, UK), with a 150 N calibrated load cell. At each loading step, the WAXS detector was translated out of the beam to expose the X-ray Eye detector in the downstream. The X-ray Eye detector was initially used by opening the slits to take the pictures of samples by radiography technique. Then the slits were closed and the SAXS detector was translated laterally and exposed to the beam to collect SAXS pattern. To record both WAXS and SAXS patterns at each scanning location, the WAXS detector was then translated laterally to the beam position to collect the WAXS pattern after each SAXS pattern collection.

### Data interpretation

Radiographic images captured by X-ray Eye were processed by ImageJ software^22^. By analysing the change of the distance between the centres of the two circles along the *x* axis (shown in Fig. 1) from a series of images taken and recorded during tensile loading, the macroscopic strain evolution can be easily determined.

Quantitative interpretation of SAXS patterns provides insight into nano-structure changes in the reciprocal space. In detail, the 2D SAXS patterns were processed by caking (that is, binned in the radial-azimuthal coordinates) within the range of 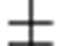
10° along the *x* and *y* directions under laboratory coordinate (see Fig. 1), and the caked images were converted into 1D radial plots of intensity (*I*(*q*)~*q*) using the Fit2D software package^23^. Subsequently the 1D radial plots within each sector were fitted with Gaussian curves to obtain the peak centre position. As the load increased, the shift of the peak centre position with respect to the strain-free reference point allowed the calculation of the variation of scattering vector (*q*), which indicates the structural changes at the nano-scale. Then, the structural dimension (*d*) changes can be reflected from the variation of *q*, and the strain *ε*_SAXS_ at nano-scale in the real space could be obtained.

The 2D diffraction WAXS images were processed by the same caking within a range of 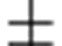
10° along both *x* and *y* directions under laboratory coordinate as that of SAXS interpretation and the converted 1D radial plots within each sector were also fitted with Gaussian curves to obtain the peak centre position. As the load increases, the shift of the peak centre position with respect to the strain-free reference condition allowed the calculation of the variation of the scattering angle (*θ*). According to Bragg’s law, the shift of peak centre position indicates how the *d*-spacing changed, that is, how the sub-nano-scale strain *ε*_WAXS_ evolved.

### Image-based FEM and FFT analysis

**Figure 7:**
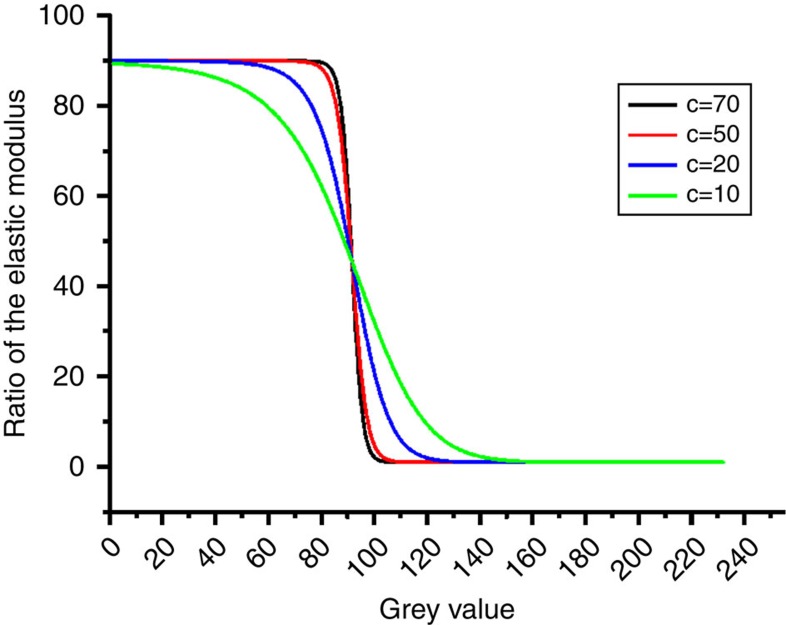
Property gradient representation. Profiles prescribed by the function given in equation (4), with the values of parameter *c* set equal to 70, 50, 20 and 10.

The finite element package ABAQUS v. 6.12 was used to simulate the *in situ* deformation of polyurethane elastomer to obtain the associated deformed shape. The first stage was to choose a RVE with 332 × 311 pixels from the TEM image of TPUs. The grey value from the images has a range {0–255} so that each pixel can be represented by eight-bit, where in detail, the grey value *g* of ‘0’ represents ‘Black’ and ‘255’ represents ‘White’. The grey value *g* was used as the principle parameter for correlating pixel brightness with material density and stiffness. The second stage was to generate a corresponding model with 332 × 311 elements in ABAQUS. Then the model was given the same colour as the greyed RVE image by importing a certain Python colour assignment script, where the range of the grey values of pixels in Fig. 4b was identified to be {76–232} as was read directly by Matlab. The assignment of the elastic modulus *E* to each element was carried out as a function of its grey-scale value *g*. The correlation was described by the ‘knee’ function of the type^24^:


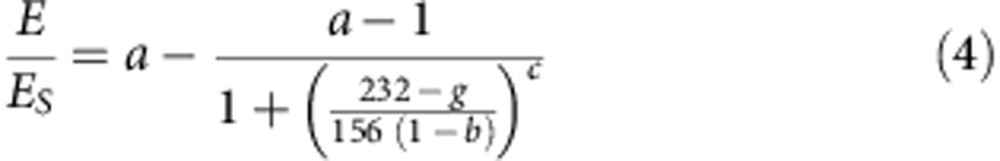


where *a* represents the ratio of the elastic modulus of the HS (darker region in RVE with lower grey values) to that of the SS (brighter region in RVE with higher grey values) and *b* describes the volume fraction of the HS. The use of this function provides the flexibility to describe, using a small number of adjustable parameters, the complex relationship between the electron density (reflected in the grey values) and the elastic modulus. The value of parameter *c* controls the steepness of the transition, as illustrated in Fig. 7. Adjustable parameters were used in the function to illustrate the ratio between extreme values of stiffness and steepness of the transition between hard and soft regions, as well as the volume fraction of the hard phase (*a*, *c* and *b*). The ratio of modulus between the hard and soft region influences the ratio 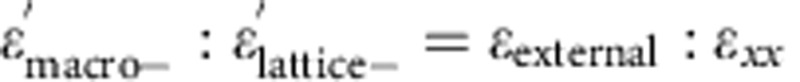. Classical composite theory, for example, the Eshelby mean field model^14^, shows that for the case of hard inclusions embedded in a soft matrix, the relationship between macro-strain and inclusion strain is inversely correlated to the corresponding ratio of moduli of the two phases. The values of parameters *b* and *c* control the steepness of the stiffness gradient across the fuzzy interface between the hard and soft regions, as well as the macro- to nano-scale strain ratio 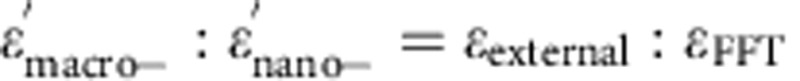. Therefore, the selection of parameters *a* in the parametric analysis carried out in the present study was relatively straightforward, while coupled adjustment of parameters *b* and *c* was required. It was possible to achieve good agreement between the ratios of strains at the three distinct length scales predicted by the model and the experimental data. This suggested that the physical mechanisms responsible for the observed strain ratios have been captured correctly. Different values of this parameter were considered. It is important to take into account the fact that it combines multiple influences. On one hand, it reflects the steepness of stiffness variation as a function of the grey-scale value *g*. On the other, it also incorporates the implied dependence of stiffness on electron density. The best fit value of this parameter is therefore dependent both on the material structure, and on the contrast setting of the TEM image. The parametric analysis carried out as part of our simulation led to the best fit value of *c*=20.

The final stage of computational analysis of the TPUs using the FEM model was to obtain the strain distribution within the material subjected to uniaxial stretching. The simulated 2D SAXS pattern was then obtained by applying 2D FFT using ImageJ software^22^ to the output from ABAQUS package that was converted to a grey-scale image for this purpose. To improve our confidence in the interpretation of results, supporting simple 1D analysis was carried out by FFT using Matlab.

## Author contributions

A.M.K. conceived the study. T.S. and N.B. performed *in situ* scattering measurement with the help of I.P.D., C.P. synthesized the materials, T.S. analysed the data and performed the simulation. T.S. wrote the manuscript with discussion and improvements from all the authors.

## Additional information

**How to cite this article:** Sui, T. *et al*. Multiple-length-scale deformation analysis in a thermoplastic polyurethane. *Nat. Commun*. 6:6583 doi: 10.1038/ncomms7583 (2015).
